# Rapid molecular diagnosis using femA mecA real-time PCR for staphylococcal bacteraemia improves early appropriate antibiotic prescribing: a randomised clinical trial

**DOI:** 10.1186/2047-2994-4-S1-O1

**Published:** 2015-06-16

**Authors:** S Emonet, PG Charles, S Harbarth, G Renzi, A Cherkaoui, M Rougemont, J Schrenzel

**Affiliations:** 1 University Hospitals Geneva, Switzerland; 2 Austin Health, Heidelberg, Australia

## Introduction

Vancomycin overuse exerts a selective pressure for VRE and VISA and has been associated with poorer outcomes than beta-lactams when treating MSSA. Rapid determination of methicillin susceptibility should be particularly helpful to spare vancomycin in a setting where MRSA prevalence is low.

## Objectives

To compare the time to targeted antimicrobial treatment of staphylococcal bacteraemia when using real-time PCR versus conventional microbiological workup. To assess the impact of PCR on the need for ICU, risk of septic complications and mortality.

## Methods

In this prospective single-centre study performed in 2012-2013, all blood culture vials positive for Gram positive cocci “in clusters" underwent *fem*A_SA, *fem*A_SE, and *mec*A real-time PCR testing for the rapid identification of staphylococcal species and methicillin-susceptibility, as well as undergoing conventional microbiology work-up. Patients who had only one blood culture bottle positive were excluded.

100 patients were randomized 1:1 for treating physicians being informed of PCR results as soon as available (Group A), or else waiting until results of conventional tests were available (Group B). Antibiotic therapy was classed as ideal (targeted), adequate or inappropriate. Time to ideal therapy was compared in both groups.

## Results

89 patients were included in the per protocol (PP) analysis, 48 in group A and 41 in group B. MRSA was identified in 7 patients, MSSA in 46 and CNS in 36. PCR results were concordant with standard microbiological testing.

On average, PCR results were available 3.2 hours after Gram stain transmission (28.9 hours for standard tests, p<0.001). 85.4% of group A patients were already receiving “ideal therapy” when the antibiogram became available (56.1% in group B, p = 0.004). Not surprisingly, *S. aureus* infections led to more complications than CNS infections (26/46 vs. 7/36; p<0.001). Septic complications did not differ between groups, nor did the need for ICU and the 28-day mortality.

## Conclusion

Rapid determination of methicillin susceptibility in staphylococcal bacteraemia drastically reduces the time to targeted antibiotherapy, thereby avoiding unneeded exposure to vancomycin.

## Disclosure of interest

None declared.

